# Siblings’ experiences of having a brother or sister with an eating disorder

**DOI:** 10.1192/j.eurpsy.2022.1492

**Published:** 2022-09-01

**Authors:** A. Heneghan, I. Manitsa, M. Livanou

**Affiliations:** 1 Kingston University London, Department Of Psychology, Kingston upon Thames, United Kingdom; 2 Kingston University, Psychology, London, United Kingdom

**Keywords:** eating disorder, experiences, Siblings

## Abstract

**Introduction:**

Despite the huge effect eating disorders (Eds) have on the lives of sufferers and their families there has been little research on the effect an ED has on siblings even though their lives are repeatedly significantly affected by the situation. It is important to gain more insight into the experiences and needs of siblings as the nature and magnitude of the effect of patients EDs on non-affected siblings is mixed in the current research.

**Objectives:**

To conduct a systematic review allowing an extensive search of the current literature to identify where the current research is lacking. Also, to highlight the need for a greater focus on the effect of EDs on siblings both in research and clinical practice.

**Methods:**

A systematic review is being conducted to gain an understanding of the gaps in the literature.

**Results:**

It is expected that the systematic review will reveal a lack of literature regarding siblings’ experiences of having a brother or sister with an ED. As well as showing the conflicting emotions felt by the siblings, both positive due to the love they feel for their sibling and negative due to the burden they feel.

**Conclusions:**

By raising awareness of the needs of non-affected siblings this research should have a notable impact on their experiences by highlighting the need for specific interventions and support services as well as education about their siblings’ ED.

**Disclosure:**

No significant relationships.

